# P-1627. The Impact of Microbiology Diagnosis Process Optimization on Clinical Outcomes and Health Economics in Hospitalized Bloodstream Infection Patients in China

**DOI:** 10.1093/ofid/ofae631.1793

**Published:** 2025-01-29

**Authors:** Genwei AI, Lu ZHAO, Zhi LI, Kunshan GUO, Ying ZHANG, Erjuan JIA, Yijun XIA

**Affiliations:** Xuchang Central Hospital, Xuchang, Henan, China; Xuchang Central Hospital, Xuchang, Henan, China; Xuchang Central Hospital, Xuchang, Henan, China; Xuchang Central Hospital, Xuchang, Henan, China; Xuchang Central Hospital, Xuchang, Henan, China; Xuchang Central Hospital, Xuchang, Henan, China; bioMérieux (Shanghai) Company, Limited, Shanghai, Shanghai, China (People's Republic)

## Abstract

**Background:**

Bloodstream infection (BSI) is one of the leading causes of morbidity and mortality worldwide. Early identification of bacteria is essential for antimicrobial stewardship and the key to improve outcomes. The aim of this study was to evaluate the impact of an optimized microbiology lab process on turnaround times, antibiotics use and economics in adult hospitalized BSI patients.

**Figure 1:**
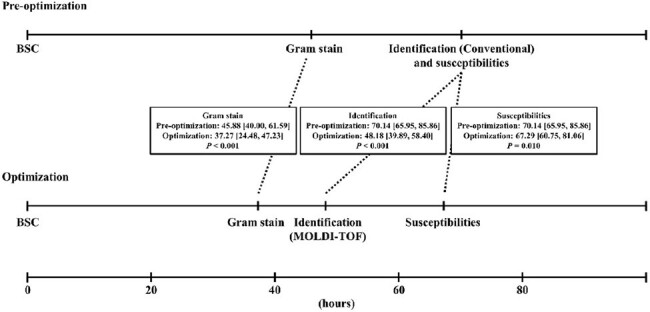
Gram stain, ID and AST reporting times between pre- and post-optimization groups.

**Methods:**

Since Oct-2021, the microbiology laboratory in Xuchang Central Hospital, Henan, China had implemented a series of improvements, including the utility of BacT/Alert Virtuo blood culture (BC) system, the new resin anaerobic/aerobic BC bottles, VITEK MS (bioMérieux), and an optimized sample reception process. Patients with positive BC results in ICU, general surgery, respiratory, hematology and emergency wards from Oct-2020 to Sep-2021 and Oct-2021 to Sep-2022 were grouped into the pre-optimization (pre-op) and the post-optimization (post-op) groups, respectively.

**Table 1:**
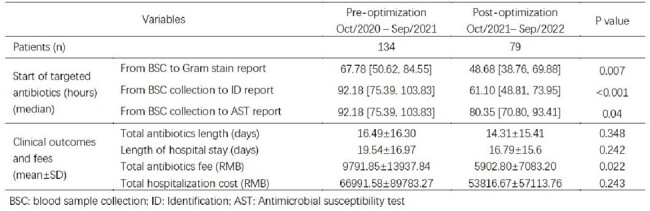
The impact of optimized microbiology lab process

**Results:**

A total of 134 patients were included in the pre-op group, 79 patients were included in the post-op group. There were no differences in baseline characteristics. Compared to pre-op group, the Gram stain reporting time of post-op group decreased significantly (37.27 vs 45.88h, p=0.001). The use of VITEK MS allowed a faster ID report, which significantly reduced the ID reporting time (48.18 vs 70.14h, p< 0.001) compared to pre-op group (Figure 1). These faster reports resulted in rapid targeted antibiotics use. The time from blood sample collection to targeted antibiotic therapy within 48h of each report (Gram stain, ID and AST) was much shorter in post-op group (Table 1). In post-op group, although the decreases of total antibiotics days and length of hospital stay didn’t show significance, the total antibiotics fee was significantly reduced (5902.80 vs 9791.85 RMB, p=0.022). Such fee saving possibly contributed in the saving of total hospitalization cost in post-op group (53816.67 vs 66991.58, P=0.243).

**Conclusion:**

Optimization of the microbiology lab process resulted in significant decreases in reporting time and ensured faster targeted antibiotic therapy in hospitalized BSI patients. Such improvements might potentially bring positive impact on antimicrobial stewardship, clinical outcomes and health economics.

**Disclosures:**

**All Authors**: No reported disclosures

